# Association of Mobile Instant Messaging Chat Group Participation With Family Functioning and Well-Being: Population-Based Cross-sectional Study

**DOI:** 10.2196/18876

**Published:** 2021-03-15

**Authors:** Sheng Zhi Zhao, Tzu Tsun Luk, Ningyuan Guo, Man Ping Wang, Agnes Yuen Kwan Lai, Bonny Yee Man Wong, Daniel Yee Tak Fong, Sophia Siu Chee Chan, Tai Hing Lam

**Affiliations:** 1 School of Nursing The University of Hong Kong Hong Kong China (Hong Kong); 2 School of Public Health The University of Hong Kong Hong Kong China (Hong Kong)

**Keywords:** mobile instant messaging, chat groups, family communication, family well-being, family functioning

## Abstract

**Background:**

Convenient and quality family communication improves family functioning and well-being. Using mobile instant messaging (IM) for family communication is increasingly popular, but its association with family functioning and family well-being has not been reported.

**Objective:**

The aim of this study was to examine the association of the use of family IM chat groups with family functioning and well-being, and the mediating effect of family communication quality among Chinese adults in Hong Kong.

**Methods:**

We analyzed data from the Family and Health Information Trend Survey (FHInTS), a territory-wide, probability-based telephone survey conducted in 2017. The quality of family communication, family functioning, and well-being was assessed using the Family Communication Scale; Family Adaptation, Partnership, Growth, Affection, and Resolve (APGAR) Scale; and Family Well-Being Scale (family heath, harmony, and happiness), respectively. Respondents also reported the number of family IM chat groups (0, 1, 2, ≥3), and numbers of IM messages received (<1, 1-2, 3-10, 11-20, >20) and sent (<1, 1-2, 3-10, 11-20, >20) daily. The frequency of family IM chat interaction (range 0-8) was calculated by combining the number of messages received from and sent to the family IM chat groups daily. Covariates included sociodemographic characteristics and the frequency of family face-to-face communication (often, sometimes, seldom, or never). Data were weighted by sex, age, and education of the general population. Adjusted β coefficients of family functioning and well-being in relation to having a family IM chat group, and numbers of messages received and sent were calculated. The mediation effects of family communication on these associations were assessed, controlling for the covariates.

**Results:**

A random sample of 1638 Chinese adults (45.6% men; 78.1% aged 25 to 64 years) were interviewed (response rate: 74.4%). Female, younger age, being married or cohabiting, higher education, higher income, better family functioning, and well-being were associated with having at least one family IM chat group (all *P*<.01). Higher scores of family communication, family APGAR, and family well-being were associated with having more family IM chat groups and more messages received from and sent to family IM chat groups daily (all *P* for trend <.01). More frequent family IM chat interaction was associated with higher scores of family communication, family APGAR, and family well-being (β=.16-.83, all *P* for trend <.001). The associations of family IM chat interaction with family functioning and well-being were moderately (51.0%-59.6%) mediated by family communication.

**Conclusions:**

Use of a family IM chat group was associated with higher family functioning and well-being, and the association was partially mediated by family communication.

## Introduction

Family functioning includes the interactions and relationships, adaptability, organization, and communication of the family environment, and is the foundation for the physical and psychosocial well-being of family members [1,2]. Family well-being, usually conceptualized as “family life satisfaction,” is the keystone of a harmonious society [3]. From a macro perspective, social resources, including income, time, and psychological and social capital, are determinants of family functioning and well-being [4]. Individually, spending time and having quality communication with family members improves family well-being, as this allows family members to connect, achieve fulfillment, and express and share attitudes and values. Higher quality family communication improves cardiovascular health, immune system, subjective well-being, and quality of life [5,6]. Having poor family communication and relationships increase the risks of depression, anxiety, loneliness, substance abuse, and other psychological distress [7,8].

The development of information and communication technologies (ICTs) has largely transformed interpersonal communication [9]. Advanced ICT tools in smartphones, such as voice messages, multimedia messages, and video calls, have facilitated the rapid exchange of information or affect [10]. However, inappropriate use of ICTs has led to a wide array of psychological problems, including decline in the size of social circles and life satisfaction, and an increase in depression and loneliness [11]. Specifically, entertainment usage, especially gaming, is associated with emotional exhaustion, function impairment, and relationship disruption [12]. A poor perceived well-being can also result from a perceived waste of time in excessive smartphone use [13]. Relationship fulfillment is usually acquired from achieving common goals or relating to others. As the most important social tie with effort to maintain, family cohesion and satisfaction require interactions with both high quantity and quality. Under the current busy social context, communication through a smartphone, including phone calls or text messages, has been associated with increased family functioning and well-being, presumably via a positive and effective experience of interactive connection [14-16].

Face-to-face communication has remained the dominant mode of family interaction [17]. Telephone, as a traditional means of remote communication, provides instant feedback across geographical distance. Instant messaging (IM) apps (eg, WhatsApp, WeChat), with access regardless of time, distance, and cost restrictions, may function as better substitutes when face-to-face communication is hard to achieve [18]. We previously reported that more than half of the Hong Kong population used IM for family communication in the 2012 household survey [19]. A higher well-being level was observed among families using smartphones for communication [19]. Long working hours have left little time for family gatherings. The increase in smartphone-mediated communication such as mobile voice and video calls is related to closer and higher-quality relationships, which are in turn related to greater life satisfaction and well-being [20]. IM features help to maintain real-time bonding and bridge social capital, which can facilitate communication for higher perceived well-being [19,20]. A group chat allows for all sources of information to be synchronously exchanged among three or more participants, and is now commonly used among classmates, coworkers, friends, and family members [21]. As a common platform for family life information sharing, an IM chat group can strengthen family management (eg, parenting, childcare) and health information transfer for improving family health [22,23].

Positive communication experiences are critical for the adaptive functioning of family relationships by buffering against family problems and difficulties [14]. Chinese culture values family communication as an important embodiment of family cohesion [24], which is considered to be vital for the family harmony, happiness, and health (3Hs) that underlie family well-being [3,25]. Lack of positive interaction, in conjunction with a stressful work life, can be detrimental to family well-being [26]. In Hong Kong, smartphone penetration reached 88.6% in 2017 and almost all internet users (98.1%) use smartphones for communication [27]. As a new approach for family communication, there is a lack of research to determine whether a family group chat is associated with well-being. Most of the recent studies in this field have focused on IM use among teenagers and adolescents with a small sample size [28,29]; however, IM users are increasing rapidly in all age groups [27]. Therefore, the aim of this study was to examine the associations of family chat group usage with family functioning and well-being in Hong Kong adults. We also tested whether the above associations are mediated by improved quality of family communication.

## Methods

### Sampling

We used data from the latest phase of the Hong Kong Family and Health Information Trends Survey (FHInTS) conducted from February to May 2017 under the Hong Kong Jockey Club FAMILY project. The FHInTS was a probability-based telephone survey that aimed to assess the use of ICTs in relation to family relationships in the Hong Kong Chinese population. Details of the research design have been reported elsewhere [19,30,31]. The Public Opinion Programme (POP) of the University of Hong Kong, one of the most reputable survey agencies in Hong Kong, conducted the telephone interviews. Respondents were Hong Kong residents aged 18 years or above who could speak Cantonese. All interviews were conducted by trained interviewers using a computer-assisted telephone interviewing system. POP adopted a two-stage random sampling method. First, the residential telephone directories that covered about 76% of Hong Kong residents [32] were used to generate a list of randomized household telephone numbers (seed numbers) by a computer program. To capture the unlisted telephone numbers, plus or minus one or two of the last digits of the seed numbers were used to generate new numbers for random dialing. Invalid household numbers and nonresponses (after 5 calls at different times and days of the week) were excluded. In the second stage, the “next birthday” rule was used to pick one eligible household resident with the closest next birthday to the interview dates as the respondent for the survey. Of the 4054 respondents who completed the interview (response rate: 74.4%), a subset of 1638 (40.4%) respondents were randomly selected to answer questions on the use of a family IM chat group, family communication, family functioning, and family well-being. Ethical approval was granted by the Institutional Review Board of the University of Hong Kong/Hospital Authority Hong Kong West Cluster. Verbal informed consent was obtained and recorded verbatim in the telephone survey.

### Measurement

The interviewers read out the definition of family (ie, family members who are related through biological, marital, cohabitation, or emotional bonding) before the interview. To assess the use of a family IM chat group, the respondents were asked “Do you have a family group of 3 or more persons using IM such as WhatsApp and WeChat group chat?” with response options of “Yes” and “No.” Those who responded “Yes” were further asked about the number of family IM chat groups they have (1, 2, 3, or >3) and the number of messages they receive from and send daily in the family IM chat groups separately with responses categorized into “None,” “1-2 messages,” “3-10 messages,” “11-20 messages,” and “Over 20 messages.”

The quality of family communication was measured by the Family Communication Scale (FCS) [33], which consists of 10 items on a one-dimensional scale focusing on positive family communication skills between family members, such as clear and congruent messages, empathy, supportive phrases, and effective problem-solving skills. The FCS aims to gather important aspects of family communication, including the level of openness and honesty to exchange ideas, emotional tone of the interactions, and affections and concerns for each other. Each item is scored on a Likert scale of 1 (strongly disagree) to 5 (strongly agree). All items are summed to give a total score of 10 to 50, with a higher score indicating better family communication. The internal consistency of the FCS in this study was excellent (Cronbach α=.90).

The Family Adaptation, Partnership, Growth, Affection, and Resolve (APGAR) scale consists of five questions assessing the frequency of feeling satisfied with five family functioning parameters on a 3-point Likert scale of 0 (hardly ever) to 2 (almost always) [34]. The statements used in the scale concerned the emotion expression, communicative, and interactive relationships between the respondents and their family, such as “You were satisfied with the care of your family when you had different emotions (including happiness, anger, sadness).” The Chinese version of the Family APGAR has been widely used in the Hong Kong population [35] and the Cronbach α in this study was good (.86).

The Family Well-Being Scale developed based on our qualitative interviews under the FAMILY project [3,36] consists of three items of family 3Hs, each measured on a scale of 0 to 10 with a higher score indicating better family 3Hs [3,37]. A composite score of family well-being (range 0-10) was the average of the family 3Hs score. The internal consistency of the 4 items was good with a Cronbach α of .89 in this study.

Sex, age, marital status (never been married, married or cohabitating, divorced or separated, and widowed) and socioeconomic status (SES), including education attainment (primary or below, secondary, or tertiary or above) and monthly household income (in Hong Kong dollars [HK $], in which US $1=HK $7.80: ≤9,999, 10,000-19,999, 20,000-29,999, 30,000-39,999, ≥40,000, and unstable) of the respondents were recorded. We also collected data on the frequency of face-to-face family communication (often, sometimes, seldom, or never).

### Statistical Analysis

The χ^2^ test was used to compare sociodemographic differences and family relationship measurements between family chat group users and nonusers. To improve the representativeness of the sample, all data were weighted according to provisional figures obtained from the Census and Statistics Department on the sex-age distributions of the Hong Kong general population in 2015 and the education attainment distribution in 2011. The mean scores of the FCS, Family APGAR, and Family Well-Being Scale were compared between respondents with or without family IM chat groups and according to usage of the family IM chat group. Multivariable linear regressions were used to calculate the regression coefficient (β) of family communication, family functioning, and family well-being in relation to the use of the family IM chat group, adjusting for age, sex, marital status, and SES (model 1). In model 2, we additionally adjusted for the frequency of face-to-face family communication. We created a composite variable for the frequency of family IM chat interaction (range 0-8) by combining the variable of the number of messages received from and sent to the family IM chat groups daily for a mediation test. The Baron and Kenny approach was used to examine the mediating effect of family communication on the associations of family IM chat interaction with family functioning and well-being [38]. The Sobel test was used to determine whether the mediating (indirect) effect was significant. Bootstrapping with 1000 replications was used to calculate the 95% CI of the indirect effect. All analyses were performed using STATA version 15.0 (StataCorp LP, College Station, TX, USA). A two-sided *P* value<.05 was considered statistically significant.

## Results

Of the 1638 respondents, just under half were men and the majority were aged 25 to 64 years. Most of the respondents were married or cohabitating, attained secondary or higher level of education, and had a monthly household income of more than HK $30,000 (Table 1). Almost all respondents communicate with family members (98.6%, 1341/1360), with the great majority often communicating face-to-face. Bivariate analysis showed that respondents of female sex, younger age, married or cohabitating, with higher education, higher income, better family functioning, and better well-being were associated with having at least one family IM chat group (all *P*<.01). The frequency of family face-to-face communication was not associated with the use of family IM chat groups (*P*=.14).

Most respondents (72.0%, 1180/1638) had at least one family IM chat group. Among those who have family IM chat groups, 92.0% (1083/1177) and 83.7% (987/1179) of the respondents received and sent at least one message daily, respectively (Table 2). Compared with having no family IM chat groups (n=458), having 3 family IM chat groups was associated with higher mean scores (Multimedia Appendix 1), and was significantly associated with a higher FCS level (mean 37.3 vs 39.5), family APGAR (mean 5.4 vs 7.0), and family well-being (mean 7.1 vs 7.8) controlling for sociodemographic factors (Table 2). Respondents with more family IM chat groups and who received or sent more messages in family IM chat groups were positively associated with a better quality of family communication, family APGAR, and family well-being with dose-response associations (all *P* for trend <.001). More frequent IM chat interactions (received and sent messages combined) was significantly associated with increased FCS, family APGAR, and family well-being levels compared with those of respondents reporting no use. The associations remained robust after additionally adjusting for the frequency of family face-to-face communication (Table 2).

The effect of a greater number of family IM chat groups, received messages, sent messages, and chat interactions (received and sent messages combined) on family functioning and family well-being was largely attenuated after adjusting for FCS (Table 3), providing support for FCS as a mediator. A significant mediation effect of FCS on the above associations (*P* for Sobel test <.001) was found independent of the frequency of family face-to-face communication. Family IM chat interaction accounted for 23.0% of the total effect on family APGAR, and 59.6% of the total effect was mediated by FCS. Similarly, FCS accounted for 51.0% of the 18% total effect of family IM chat interaction on family well-being (*P* for Sobel test <.001).

**Table 1:** Sociodemographic characteristics according to family instant message chat group use.

Characteristic			No family chat group (n=458), n (%)		Had family chat group (n=1180), n (%)		*P* value		Total (N=1638)			
										Crude, n (%)^a^		Weighted, n (%)^b^
**Sex**							<.001					
	Male			218 (47.6)		423 (35.9)				641 (39.1)		747 (45.6)
	Female			240 (52.4)		757 (64.2)				997 (60.9)		891 (54.4)
**Age (years)**							<.001					
	18-24			54 (11.8)		156 (13.2)				210 (12.8)		170 (10.4)
	25-44			50 (10.9)		241 (20.4)				291 (17.8)		662 (40.4)
	45-64			137 (29.9)		525 (44.5)				662 (40.4)		618 (37.7)
	≥65			217 (47.4)		258 (21.9)				475 (29.0)		188 (11.5)
**Marital status**							.006					
		Never been married	115 (25.1)		307 (26.0)				422 (25.7)		293 (17.9)	
		Married or cohabitating	276 (60.3)		770 (65.3)				1046 (63.9)		1125 (68.7)	
		Divorced or separated	22 (4.8)		33 (2.8)				55 (3.4)		60 (3.7)	
		Widowed	45 (9.8)		70 (5.9)				115 (7.0)		160 (9.8)	
**Education attainment**							<.001					
		≤Primary	122 (26.6)		123 (10.4)				245 (15.0)		267 (16.3)	
		Secondary	198 (43.2)		515 (43.6)				713 (43.5)		837 (51.1)	
		≥Tertiary	138 (30.1)		542 (45.9)				680 (41.5)		534 (32.6)	
**Monthly household income (HK$)^c^**							<.001					
		≤9999	128 (28.0)		138 (11.7)				266 (16.2)		170 (10.4)	
		10,000-19,999	85 (18.6)		145 (12.3)				230 (14.0)		277 (16.9)	
		20,000-29,999	62 (13.5)		225 (19.1)				287 (17.5)		347 (21.2)	
		30,000-39,999	41 (8.9)		164 (13.9)				205 (12.5)		241 (14.7)	
		≥40,000	84 (18.3)		372 (31.5)				456 (27.8)		447 (27.3)	
		Unstable	58 (12.7)		136 (11.5)				194 (11.8)		156 (9.5)	
**Frequency of family face-to-face communication (n=1360)**							.14					
		Often	207 (68.5)		598 (75.1)				959 (70.5)		976 (71.8)	
		Sometimes	68 (22.5)		149 (18.7)				280 (20.6)		262 (19.3)	
		Seldom	23 (7.6)		43 (5.4)				93 (6.8)		102 (7.5)	
		Never	4 (1.3)		6 (0.8)				28 (2.1)		19 (1.4)	

^a^Sample size varied because of missing values.

^b^Weighted by the sex-age distributions of the Hong Kong general population in 2015 and the education attainment distribution in 2011.

^c^HK$: Hong Kong dollars; HK $7.8=US $1.

**Table 2:** Association of the number of family instant message (IM) chat groups (N=1638) and use (N=1180) with family communication, family functioning, and family well-being.

Variable		Respondents, n (%)		Family communication quality (10-50), adjusted β (95% CI)				Family functioning^a^ (0-10), adjusted β (95% CI)				Family well-being (0-10), adjusted β (95% CI)			
					Model 1^b^		Model 2^c^		Model 1		Model 2		Model 1		Model 2
**Number of family IM groups**															
	0		458 (28.0)		Reference		Reference		Reference		Reference		Reference		Reference
	1		356 (21.7)		.82 (–.76 to 2.42)		.64 (–.88 to 2.15)		.70 (.17 to 1.24)^**^		.20 (–.55 to .96)		.27 (.04 to .49)^*^		.11 (–.16 to .38)
	2		273 (16.7)		1.02 (–.67 to 2.72)		.86 (–.75 to 2.48)		.52 (–.06 to 1.10)		.19 (–.62 to .99)		.39 (.14 to .64)^**^		.21 (–.09 to .51)
	≥3		551 (33.6)		2.39 (1.01 to 3.76)^***^		2.04 (.72 to 3.35)^**^		1.40 (.91 to 1.89)^***^		1.09 (.43 to 1.75)^***^		.61 (.40 to .82)^***^		.39 (.15 to .64)^**^
	*P* for trend				<.001		.002		<.001		.001		<.001		.001
**Number of received IM from family chat groups/day**															
	<1		94 (8.0)		Reference		Reference		Reference		Reference		Reference		Reference
	1-2		338 (28.7)		3.81 (1.21 to 6.40)^**^		2.73 (.22 to 5.24)^*^		1.32 (.44 to 2.21)^**^		.41 (–.84 to 1.66)		.25 (–.10 to .59)		.03 (–.36 to .43)
	3-10		533 (45.3)		4.32 (1.85 to 6.78)^***^		3.35 (.97 to 5.73)^**^		1.74 (.89 to 2.59)^***^		.84 (–.35 to 2.03)		.57 (.24 to .90)^***^		.35 (–.03 to .72)
	11-20		110 (9.4)		6.76 (3.53 to 10.01)^***^		5.39 (2.25 to 8.52)^***^		2.30 (1.26 to 3.33)^***^		1.17 (–.39 to 2.72)		.81 (.40 to 1.23)^***^		.78 (.28 to 1.28)^*^
	>20		102 (8.7)		6.28 (3.23 to 9.32)^***^		4.96 (2.01 to 7.91)^***^		2.72 (1.66 to 3.79)^***^		1.64 (.17 to 3.11)^*^		.93 (.51 to 1.36)^***^		.79 (.30 to 1.27)^*^
	*P* for trend				<.001		<.001		<.001		.007		<.001		<.001
**Number of sent IM in family chat groups/day**															
	<1		192 (16.3)		Reference		Reference		Reference		Reference		Reference		Reference
	1-2		503 (42.7)		2.70 (.86 to 4.53)^**^		2.46 (.70 to 4.22)^**^		.80 (.17 to 1.43)^*^		.68 (–.19 to 1.56)		.44 (.19 to .69)^***^		.39 (.10 to .68)^**^
	3-10		410 (34.8)		3.83 (1.90 to 5.75)^***^		3.13 (1.27 to 4.99)^***^		1.35 (.70 to 2.01)^***^		1.11 (.19 to 2.03)^*^		.68 (.42 to .94)^***^		.57 (.27 to .87)^***^
	11-20		49 (4.2)		5.93 (2.61 to 8.19)^***^		5.32 (2.24 to 8.40)^***^		1.71 (.57 to 2.86)^***^		1.11 (–.41 to 2.63)		1.24 (.77 to 1.72)^***^		1.34 (.79 to 1.89)^***^
	>20		25 (2.0)		4.34 (.11 to 8.57)^*^		3.50 (–.55 to 7.55)		2.90 (1.33 to 4.47)^***^		2.96 (.95 to 4.97)^**^		.78 (.15 to 1.42)^**^		.62 (–.09 to 1.33)
	*P* for trend				<.001		.001		<.001		.002		<.001		<.001
Frequency of family IM chat interaction^d^					.83 (.47 to 1.19)^***^		.69 (.34 to 1.04)^***^		.36 (.23 to .48)^***^		.27 (.10 to .44)^**^		.16 (.11 to .21)^***^		.15 (.09 to .21)^***^

^a^Family functioning assessed on the Family Adaptability, Partnership, Growth, Affection, and Resolve (APGAR) scale.

^b^Regression model 1: adjusted for sex, age, education attainment, family income, and marital status.

^c^Regression model 2: additionally adjusted for the frequency of face-to-face family communication (often, sometimes, seldom, or never).

^d^Composite variable, frequency of family IM chat interaction (range 0-8), sum of the number of messages received from IM chat groups and number of messages of sent in IM chat groups per day.

^*^P<.05; ^**^P<.01; ^***^P<.001.

**Table 3:** Adjusted indirect, direct, and total effects of the number of family instant message (IM) chat groups (N=1638) and use (N=1180) on family functioning and family well-being mediated by family communication using the Sobel test.

Variable		Number of family IM chat groups		Number of received IM from family chat groups/day		Number of sent IM in family chat groups/day		Frequency of family IM chat interaction^a^	
**Family functioning**									
	Total effect, adjusted β^b^ (95% CI)		.33 (.13 to .54)^**^		.34 (.05 to .63)^*^		.45 (.12 to .78)^**^		.23 (.06 to .40)^**^
	Indirect effect (through mediator), adjusted β (95% CI)		.14 (.04 to .23)^**^		.22 (.08 to .36)^**^		.23 (.09 to .38)^**^		.14 (.06 to .22)^***^
	Direct effect (without mediator), adjusted β (95% CI)		.20 (–.01 to .40)		.12 (–.16 to .39)		.22 (–.09 to .52)		.09 (–.06 to .24)
	Proportion of total effect mediated (%)		40.4		64.9		51.8		59.6
**Family well-being**									
	Total effect, adjusted β (95% CI)		.14 (.03 to .25)^*^		.31 (.16 to .46)^***^		.31 (.14 to .49)^***^		.18 (.10 to .27)^***^
	Indirect effect, adjusted β (95% CI)		.10 (.03 to .16)^**^		.15 (.06 to .24)^***^		.16 (.06 to .25)^***^		.09 (.04 to .14)^***^
	Direct effect, adjusted β (95% CI)		.04 (–.05 to .14)		.16 (.04 to .28)^**^		.15 (.02 to .29)^*^		.09 (.02 to .15)^**^
	Proportion of total effect mediated (%)		68.6		48.4		50.6		51.0

^a^Composite variable, frequency of family IM chat interaction (range 0-8), sum of the number of messages received from IM chat groups and number of messages sent in IM chat groups per day.

^b^Regression model 2: adjusted for sex, age, education attainment, family income, marital status, and frequency of family face-to-face communication.

^*^P<.05; ^**^P<.01; ^***^P<.001.

## Discussion

To our knowledge, this is the first study to report the associations of family IM chat groups with family communication, functioning, and well-being. Family interaction has been strongly associated with family relationships and well-being [5,39]. Our findings are in line with previous findings on the use of IM for family communication predicting greater positive relations and well-being [19]. Three-quarters (73.3%) of the respondents had at least one family IM chat group and almost all (93%) of the family IM chat group users received or sent at least one message daily. Respondents with higher education levels, higher household income, and younger age were more likely to use IM for family communication, which might be explained by their higher accessibility and acceptance of new ICTs [19]. The associations remained robust after adjusting for sociodemographic factors and the frequency of family face-to-face communication. Having at least one family chat group was significantly associated with better family functioning and well-being. A dose-response effect of having more family chat groups was found with better family communication and relationships, in concordance with the Chinese “big family” concept, which values the togetherness, cohesiveness, and harmony of the family over individual expression [40]. The adoption of family groups also responds to a deep cross-cultural need to strengthen and maintain family intimacy, and to establish a coherent identity, especially with geographically separated families [40]. Although the information on which family members were included in the family groups was not collected, our results indicate that setting up family chat groups may increase family cohesion and impose a better family relationship.

The number of messages received (21.9% received over 10 messages daily) exceeded the number of messages sent (8.0% sent over 10 messages daily), likely because messages sent in group chats were received by more than one recipient. Respondents with frequent group interaction might have more concerns over family issues, be more eager to share family life information, and have better management of family relationships, thereby improving family well-being [14]. Prior studies have reported that traditional methods of communication (face-to-face and phone calls) were strongly associated with family well-being [19,22]. Increasing reliance on ICTs in recent years has changed and continues to transform the ways families interact, exchange information, and communicate [41]. The steep growth of IM users and the proliferation of IM app features (eg, video calls, file transfer, news sharing, emoji) have provided the most convenient communication ecosystem for society as a whole [16,29].

The association between family group chat and family well-being was partially mediated by quality communication. Maintaining family well-being is increasingly challenging in modern societies with busy lifestyles, long work hours (Hong Kong residents work an average of 45 hours a week), and poor work-life balance [42]. People who can utilize effective communication to maintain harmonious marital and family relationships are often healthier and happier than those who are in turbulent family relationships [39]. IM chat adds no time demands for gathering, and may provide opportunity to balance work and life conflicts. Group chats simulate a gathering environment where users can effectively share family information, deal with family problems, and provide support, even from a far distance. Our results, if confirmed by prospective data, could provide solid evidence that the use of family chat groups should be encouraged, especially under a busy society context.

Although specific information topics shared in family groups were not determined in this study, IMs in family chat groups may tend to be tailored to the needs of families rather than to individuals as compared to one-on-one conversations. Family members might be more inclined to share positive family information, express concern and affection, and to respond to and resolve personal or family problems in the chat groups, which are considered the main aspects of family communication quality, and are closely associated with strengthened family bonds and psychological support [39]. Gratitude and happiness expressed during family activities are positively associated with family communication and well-being [43]. Using a synchronous communication platform, IM group chat could facilitate family information exchange [22], thereby contributing to improved family functioning and well-being. Future qualitative studies may consider capturing the detailed content of the group conversation that contributes to family functioning and well-being for guiding quality family communication.

Many studies have focused on adolescents or young adults with respect to the use of IM and well-being owing to their higher tendency of use [44]; however, a huge growing trend among adults and elderly users of IM also requires research to understand the possible individual and family implications [45]. In this sample of Chinese adults, almost half (45%) of older adults (aged 65 or above) had no family chat group. One explanation is their perceived and practical access barriers to new technologies [46]. Lack of cognitive skills, information literacy, and social support in the elderly are also responsible for communication inequalities in a digitally dense society [46,47]. We found no interactions between the younger (aged 18-64 years) and older (aged over 65 years) adults on the association between family IM group chat with family communication and family relationships (see Multimedia Appendix 2), suggesting that communication with family members through chat groups equally improved perceived family relationships in the elderly as in the younger adults. Loneliness, isolation, and suicide attempts are invading the psychological health of the elderly, representing a huge public health concern [48,49], especially for aging societies such as Hong Kong [50]. Involving the elderly to use ICTs to communicate could be practical and effective in maintaining family connection; preventing family and social life isolation; and sustaining a higher level of mental, family, and social well-being.

This study has several limitations. First, landline telephone directories were used to sample the potential respondents, which excluded families with only mobile phones. Our recent landline and online (randomly sampled from a population-representative mobile panel) survey found similar characteristics in terms of sex, age, socioeconomic background, and information seeking [51]. Mobile phone and online surveys are therefore needed to complement the findings. Second, recall bias in reporting the number of family chat groups, and messages received and sent in the groups daily could not be excluded. Third, due to the cross-sectional survey design, we cannot rule out residual confounding or reverse causality. Therefore, prospective studies are needed to further confirm the associations and test for the mediation effect we observed. Indeed, families with better family relationships might tend to use IM group chat more frequently to maintain contact. Nevertheless, the association remained robust after controlling for the frequency of face-to-face communication. Most studies on IM use have focused on teenagers and adolescents owing to their higher acceptance of new technologies and lower self-control to behavioral addiction. We used simple measurements for the use of family chat groups, and the content of messages was not assessed, which warrants future investigation. We only investigated the adult population, who have more concerns and tend to pay much more attention to family relationships; thus, whether the family IM group chat equally benefits the younger generation remains unclear. Family health, harmony, and happiness have been identified as determinants of family well-being in Chinese culture [3,25]; however, the perceptions of family well-being might be different in other countries, which affects the generalizability of our findings. The validity of family well-being was indirectly supported by the consistent results with the FCS and family functioning scale, which are validated scales frequently used in Western countries.

In summary, family IM chat use was associated with higher family functioning and well-being, and the association was partially mediated by family communication.

## Appendix

Multimedia Appendix 1 Means and associations of the number of family instant message chat groups (N=1638) and use (N=1180) with family communication, family functioning, and family well-being.

Multimedia Appendix 2 Moderating effects of age on the association of the number of family instant message chat groups (N=1638) and use (N=1180) with family functioning and well-being.

## Abbreviations

3Hs: perceived family harmony, happiness, and health
APGAR: Adaptation, Partnership, Growth, Affection, and Resolve
FCS: Family Communication Scale
FHInTS: Family and Health Information Trends Survey
HK $: Hong Kong dollars
ICT: Information and communication technologies
IM: instant messaging
POP: Public Opinion Programme
SES: socioeconomic status
