# Supplementary material for: Association of Mobile Instant Messaging Chat Group Participation With Family Functioning and Well-Being: Population-Based Cross-sectional Study
Source: J Med Internet Res. 2021 Mar 15;23(3):e18876. doi: 10.2196/18876 (PMC8074847; doi:10.2196/18876)
Supplement: Multimedia Appendix 1 [file jmir_v23i3e18876_app1.docx]

**Mean and association of the number of family instant message chat group (N=1638) and use (N=1180) with family communication, family functioning, and family well-being.**

|  | **Family communication quality (10-50)** | | **Family functioning^a^ (0-10)** | | **Family well-being (0-10)** | |
| --- | --- | --- | --- | --- | --- | --- |
|  | **Mean (SD)** | **Crude β** | **Mean (SD)** | **Crude β** | **Mean (SD)** | **Crude β** |
| **No. of family IM^b^ chat groups** | | |  |  |  |  |
| 0 | 37.3 (6.2) | 0 | 5.4 (3.2) | 0 | 7.1 (1.8) | 0 |
| 1 | 37.7 (7.1) | 0.35 (–1.18 to 1.88) | 6.1 (3.2) | 0.76 (0.24 to 1.28)^**^ | 7.4 (1.7) | 0.24 (0.02 to 0.47)^*^ |
| 2 | 37.8 (5.9) | 0.49 (–1.11 to 2.10) | 6.0 (2.9) | 0.59 (0.03 to 1.15)^*^ | 7.5 (1.5) | 0.38 (0.13 to 0.62)^**^ |
| ≥3 | 39.5 (5.8) | 2.18 (0.86 to 3.50)^***^ | 7.0 (3.0) | 1.60 (1.13 to 2.06)^***^ | 7.8 (1.4) | 0.66 (0.46 to 0.86)^***^ |
| *P* for trend |  | .001 |  | <.001 |  | <.001 |
| **No. of received IM from family chat groups/day** | | |  |  |  |  |
| <1 | 34.5 (9.4) | 0 | 4.8 (3.3) | 0 | 7.1 (1.9) | 0 |
| 1-2 | 38.1 (6.4) | 3.60 (0.99 to 6.22)^**^ | 6.2 (3.0) | 1.35 (0.45 to 2.24)^**^ | 7.3 (1.6) | 0.22 (–0.12 to 0.57) |
| 3-10 | 38.8 (5.6) | 4.34 (1.85 to 6.83)^***^ | 6.6 (3.0) | 1.80 (0.94 to 2.66)^***^ | 7.7 (1.5) | 0.57 (0.24 to 0.90)^***^ |
| 11-20 | 41.1 (6.2) | 6.63 (3.36 to 9.89)^***^ | 7.1 (2.8) | 2.35 (1.30 to 3.39)^***^ | 7.9 (1.2) | 0.79 (0.38 to 1.21)^***^ |
| >20 | 40.2 (5.4) | 5.69 (2.67 to 8.71)^***^ | 7.4 (2.8) | 2.60 (1.53 to 3.67)^***^ | 8.0 (1.3) | 0.85 (0.43 to 1.28)^***^ |
| *P* for trend |  | <.001 |  | <.001 |  | <.001 |
| **No. of sent IM in family chat groups/day** | | |  |  |  |  |
| <1 | 36.0 (7.0) | 0 | 5.4 (3.2) | 0 | 7.1 (1.8) | 0 |
| 1-2 | 38.4 (6.3) | 2.43 (0.57 to 4.28)^*^ | 6.3 (3.1) | 0.88 (0.24 to 1.52)^**^ | 7.5 (1.5) | 0.41 (0.16 to 0.67)^***^ |
| 3-10 | 39.6 (5.7) | 3.60 (1.66 to 5.54)^***^ | 6.9 (2.9) | 1.48 (0.82 to 2.14)^***^ | 7.8 (1.4) | 0.66 (0.40 to 0.92)^***^ |
| 11-20 | 41.4 (5.5) | 5.39 (2.17 to 8.61)^***^ | 7.3 (2.8) | 1.90 (0.75 to 3.05)^***^ | 8.4 (1.1) | 1.25 (0.78 to 1.73)^***^ |
| >20 | 39.3 (5.8) | 3.32 (–0.85 to 7.48) | 8.1 (2.5) | 2.64 (1.06 to 4.21)^***^ | 7.8 (1.6) | 0.73 (0.10 to 1.35)^*^ |
| *P* for trend |  | <.001 |  | <.001 |  | <.001 |
| **Frequency of family IM chat interaction^c^** | | |  |  |  |  |
|  |  | 0.75 (0.39 to 1.10)^***^ |  | 0.35 (0.23 to 0.48)^***^ |  | 0.15 (0.10 to 0.20)^***^ |

^a^Family functioning APGAR (Adaptability, Partnership, Growth, Affection, and Resolve) scale.

^b^IM: instant message.

^c^Composite variable, frequency of family IM chat interaction (ranging from 0 to 8), sum of the variable of No. of messages received from IM chat groups and No. messages of sent in IM chat groups per day.

^*^*P*<.05; ^**^*P*<.01; ^***^*P*<.001.
