# Supplementary material for: Association of Mobile Instant Messaging Chat Group Participation With Family Functioning and Well-Being: Population-Based Cross-sectional Study
Source: J Med Internet Res. 2021 Mar 15;23(3):e18876. doi: 10.2196/18876 (PMC8074847; doi:10.2196/18876)
Supplement: Multimedia Appendix 2 [file jmir_v23i3e18876_app2.docx]

**Moderating effects of age on the association of the number of family instant message chat group (N=1638) and use (N=1180) with family functioning and well-being.**

|  | **Family functioning^a^** | | **Interaction term^b^** | **Family well-being** | | **Interaction term^b^** |
| --- | --- | --- | --- | --- | --- | --- |
|  | Adjusted β^c^ | Adjusted β^c^ | *P* | Adjusted^c^ | Adjusted β^c^ | *P* |
|  | Aged 18-64 (n=1163) | Aged 65 or above (n=475) |  | Aged 18-64 (n=1163) | Aged 65 or above (n=475) |  |
| **No. of family IM^d^ chat groups** |  |  | .22 |  |  | .44 |
| 0 | 0 | 0 |  | 0 | 0 |  |
| 1 | 0.71 (0.07 to 1.35)^*^ | 0.51 (–0.50 to 1.51) |  | 0.29 (0.02 to 0.56)^*^ | 0.28 (–0.16 to 0.72) |  |
| 2 | 0.50 (–0.17 to 1.17) | 0.34 (–0.89 to 1.58) |  | 0.48 (0.20 to 0.77)^***^ | 0.02 (–0.52 to 0.56) |  |
| ≥3 | 1.12 (0.52 to 1.72)^***^ | 2.10 (1.26 to 2.94)^***^ |  | 0.65 (0.39 to 0.90)^***^ | 0.54 (0.17 to 0.92)^**^ |  |
| *P* for tend | .001 | <.001 |  | <.001 | .01 |  |
| **No. of received IM in family chat groups/day** | (n=919) | (n=258) | .75 | (n=919) | (n=258) | .75 |
| 0 | 0 | 0 |  | 0 | 0 |  |
| 1-2 | 1.20 (0.17 to 2.22)^*^ | 1.95 (0.26 to 3.65)^*^ |  | 0.36 (–0.05 to 0.76) | –0.05 (–0.71 to 0.61) |  |
| 3-10 | 1.65 (0.66 to 2.64)^***^ | 2.08 (0.44 to 3.72)^*^ |  | 0.63 (0.25 to 1.02)^***^ | 0.42 (–0.22 to 1.06) |  |
| 11-20 | 1.99 (0.81 to 3.17)^***^ | 3.34 (1.20 to 5.48)^**^ |  | 0.91 (0.44 to 1.38)^***^ | 0.49 (–0.46 to 1.43) |  |
| > 20 | 2.52 (1.32 to 3.71)^***^ | 3.37 (0.85 to 5.90)^**^ |  | 0.96 (0.49 to 1.44)^***^ | 1.03 (–0.05 to 2.11) |  |
| *P* for tend | <.001 | .003 |  | <.001 | .008 |  |
| **No. of sent IM in family chat groups/day** | (n=921) | (n=258) | .53 | (n=921) | (n=258) | .73 |
| 0 | 0 | 0 |  | 0 | 0 |  |
| 1-2 | 0.78 (0.04 to 1.52)^*^ | 0.86 (–0.37 to 2.09) |  | 0.36 (0.07 to 0.66)^*^ | 0.65 (0.16 to 1.13)^**^ |  |
| 3-10 | 1.47 (0.71 to 2.23)^***^ | 0.85 (–0.48 to 2.17) |  | 0.63 (0.33 to 0.93)^***^ | 0.73 (0.20 to 1.27)^**^ |  |
| 11-20 | 1.70 (0.41 to 3.00)^**^ | 1.28 (–1.17 to 3.74) |  | 1.21 (0.67 to 1.75)^***^ | 1.25 (0.22 to 2.28)^*^ |  |
| >20 | 2.88 (1.30 to 4.47)^***^ | —^e^ | — | 0.73 (0.09 to 1.37)^*^ | — | — |
| *P* for tend | <.001 | .23 |  | <.001 | .003 |  |
| **More frequently family IM chat interaction^f^** | 0.34 (0.21 to 0.48)^***^ | 0.34 (0.06 to 0.62)^*^ | .92 | 0.15 (0.10 to 0.21)^***^ | 0.19 (0.07 to 0.31)^**^ | .20 |

^a^Family functioning based on the APGAR (Adaptability, Partnership, Growth, Affection, and Resolve) scale.

^b^Adjusted for sex, age, education attainment, family income, and marital status.

^c^Adjusted for sex, education attainment, family income, and marital status.

^d^IM: instant message.

^e^—: not applicable.

^f^Composite variable, frequency of family IM chat interaction (ranging from 0 to 8), sum of the variable of No. of messages received from IM chat groups and No. messages of sent in IM chat groups per day.

^*^*P* < .05; ^**^*P* < .01; ^***^*P* <.001
